# Structuring Materials to Support Student Learning: Analysis of Instructional Materials from a Professional Development Workshop

**DOI:** 10.1021/acs.jchemed.4c00783

**Published:** 2024-09-30

**Authors:** Andrew Kreps, Ian Brown, Thomas J. Wenzel, Renée Cole

**Affiliations:** † Department of Chemistry, University of Iowa, Iowa City, Iowa 52242, United States; ‡ Department of Chemistry, Bates College, Lewiston, Maine 04240, United States

**Keywords:** Chemical Education Research, Professional Development, Scaffolding, Active-Learning Activities

## Abstract

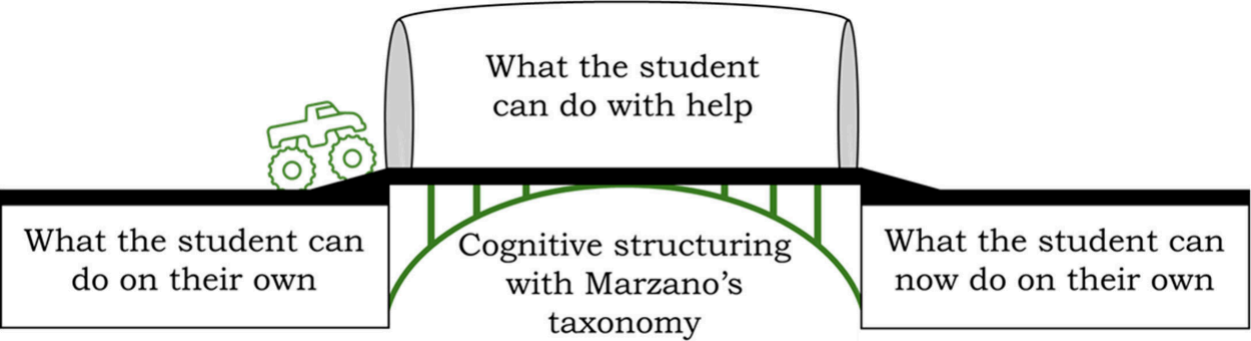

Faculty development programs play a crucial role in enhancing learning by equipping educators with the necessary skills, knowledge, and pedagogical strategies to teach more effectively. One such program is the Promoting Active Learning in Analytical Chemistry (PALAC) workshop, which aimed to educate faculty on methods to create and use active learning course materials to support students during the process of learning. This research aimed to assess the design of classroom instructional materials generated by faculty that attended the PALAC workshops. The theories of Vygotsky’s zone of proximal development and scaffolding were used as lenses to characterize the materials because they describe the benefits of providing support through the process of developing knowledge. The active learning materials were analyzed by assigning the cognitive levels of processing, as described by Marzano’s taxonomy, to all questions asked across 134 in-class activities. The use of the cognitive levels of processing allowed the researchers to gauge the presence of scaffolding by tracking how the cognitive levels of processing changed from question to question across each in-class activity. The results from this study indicate that the majority of materials provide opportunities for students to engage with higher-order questions, but there is less evidence for the effective and consistent structuring of the materials. These results have implications for future faculty development programs, suggesting the need to allot more time for faculty to practice developing effective classroom materials. In conjunction, this work demonstrates the effective use of Marzano’s taxonomy in assessing the cognitive structure of in-class activities.

## Introduction

Active learning strategies are a common topic of discussion in education research, with studies showing they can improve student learning while also reducing outcome gaps.^1,2^ With the established benefits of active learning, a “second-generation of research” began that focuses on how instructors can engage and support students in active learning. Many approaches can be taken to address these questions, from implementing learning theories like constructivism into courses to faculty development programs that aim to disseminate research-based teaching practices. One method for supporting students can be to increase the structure of a course by including graded review assignments and opportunities for in-class engagement, which have been demonstrated to increase student performance and reduce outcome gaps.^3−5^ In this article, we explore supporting student learning through the cognitive structure of in-class activities.

### Scaffolding and Cognitive Structuring

Scaffolding is a method for providing initial support to students learning new content or skills outside their current capabilities.^6,7^ The implementation of scaffolding can take many forms throughout a course and can be described by the work of Wood and Ross,^8^ who listed six functions of supporting students during the learning process: *recruitment, reduction in degrees of freedom, direction maintenance, marking critical features, frustration control, and demonstrations*. Where scaffolding differs from generally increasing course structure is that scaffolding emphasizes providing support during the learning process. For example, the *reduction in degrees of freedom* is where the number of possible actions or content needed to come to a solution is reduced or limited, and for *marking critical features*, the students are provided support through the identification of an integral part of the content that helps uncover any possible discrepancies in their thinking. These two supports highlight how scaffolding and increasing course structure can pertain to the organization and design of the overall course, but scaffolding specifically emphasizes the specific needs of students at each step of the learning process.

Scaffolding can be broadly categorized into soft and hard scaffolds.^9,10^ Soft scaffolds are dynamic and require the instructor to be actively aware of the difficulties their students are experiencing so they can provide support in real-time, while hard scaffolds are predetermined and embedded at common points of difficulty in the students’ learning. Some examples of hard scaffolds can be clicker questions, review assignments, and demonstrations, which align with similar benefits seen in studies around course structure.^3,11−14^ The research presented here focuses on hard scaffolding in the form of in-class activities. These activities provide an opportunity for faculty to develop support for students that incorporate some of the functions described by Wood and Ross, like *direction maintenance, marking critical features, and reducing the degrees of freedom*.^8^

One way to think about scaffolding through in-class activities is through the lens of cognitive structure. Tharp and Gallimore (1988) define cognitive structuring as “. . . the provision of a structure for thinking and acting” (p. 63).^15^ One method for structuring student thinking is with Marzano and Kendall’s taxonomy of educational objectives, which describes four levels of cognitive processing: *retrieval, comprehension, analysis, and knowledge utilization*, and are defined in Table 1.^16^ (See the Supporting Information for full codebook.)

**Table 1:** Cognitive Levels of Processing As Defined by Marzano and Kendall’s Taxonomy of Educational Objectives^16^

	Level of cognitive processing	Definition
Lower-order	Level 1 - Retrieval	Recalling information from permanent memory
	Level 2 - Comprehension	Identification of critical features needed to transfer knowledge from working memory to permanent
Higher-order	Level 3 - Analysis	Creating new insights from previous knowledge or using previously obtained knowledge in novel situations (focus is on the concept/skill)
	Level 4 - Knowledge Utilization	Using knowledge for novel and specific situations (focus is on the situation)

The levels of cognitive processing describe how the learner manipulates, incorporates, and uses information during the learning process. The four levels are hierarchical in nature and can be used to develop in-class materials structured according to the cognitive levels of processing. For example, the analysis level of processing requires students to apply their knowledge to generate new insights, knowledge that must be retrieved from their permanent memory and applied to a novel situation through the critical features of that knowledge to then generate new insights. This contrasts to another often-used taxonomy, Bloom’s taxonomy, which defines six levels of cognitive processing. Similar to Marzano, Bloom’s taxonomy is intended to be somewhat hierarchical in nature but has been criticized for its oversimplification and reliance on behavioral-specific goals, with studies demonstrating issues with the taxonomy’s reliability.^16−19^ The main issue with Bloom’s taxonomy, as well as others, is that a task’s difficulty is used to distinguish between the taxonomy’s levels and it only distinguishes between higher order and lower order tasks, the six levels themselves are not hierarchical despite often being represented as a pyramid. Marzano’s taxonomy corrects this issue by pulling from cognitive literature and developing its four levels of cognitive processing according to how information flows during learning.

Another benefit of using Marzano’s taxonomy is that the last two levels (analysis and knowledge utilization) align with current literature regarding higher-order thinking, where the students go beyond the common memorization-based approach and engage with information or scenarios in a more cognitively challenging way.^1^ The higher-order levels of processing are where cognitive structure becomes important because the cognitive load induced by these higher-order questions can lead to a reduction in problem-solving performance and negatively impact learning, while cognitively structuring questions can reduce the cognitive load from higher-order processing.^20−23^ By first prompting the students to recall what knowledge they will need to address a question and then identifying the critical features, we can provide the support of a *reduction in degrees of freedom* and *marking critical features* before students engage in the higher levels of processing. An example of cognitive structuring being used to develop scaffolding can be seen in the work of Toledo and Dubas, where an entire course was organized around providing cognitive structuring to students.^24^ While cognitive structuring and scaffolding are closely related, we feel it is important to acknowledge that the work presented here aims to assess the cognitive structure of in-class activities rather than scaffolding. A single activity may not fully represent the scaffolding present in a course but does provide useful insight into how cognitive structuring can be implemented to aid in the development of a scaffolded course.

### Faculty Development

Faculty development programs and workshops contribute to the continuous improvement of chemistry education, ensuring that students receive a high-quality learning experience that prepares them for future scientific endeavors. Some examples of long-running successful faculty development programs described in the literature are the Cottrell Scholars,^25^ the POGIL Project,^26^ Biology FEST,^27^ and Cutting Edge.^28^ One major challenge faculty face when adopting new teaching practices, is time.^29^ Large upfront time commitments and a lack of time spent exposed to a teaching innovation can decrease the likelihood of adoption.^30^ Some faculty development programs address the issue of time by providing participants time on task through producing materials,^31^ modeling active learning practices,^27^ and/or providing follow-up observations.^28,32^ Another similar project was Promoting Active Learning in Analytical Chemistry (PALAC). This project, which was affiliated with the Active Learning site of the Analytical Sciences Digital Library (ASDL),^33^ aimed to educate analytical chemistry faculty on the effective use of active learning techniques as well as provide long-term networking and support with other instructors. The Active Learning site of ASDL contains many of the active analytical chemistry learning materials developed during this project, which are available for free to instructors and students. The results from this project included an increase in participants’ awareness of active learning techniques and a desire to implement them into their courses.^34,35^

Although initial analysis of the workshop demonstrated increased awareness of active learning techniques, this study aimed to assess in-class activities developed by faculty that attended the PALAC workshop for the presence of cognitive structuring using Marzano’s taxonomy. The project was guided by the following research question: To what extent did participants of an active-learning faculty development program design classroom activities that were structured to provide support for higher-order levels of thinking?

## Methods

### Theoretical Framework

**Figure 1:**
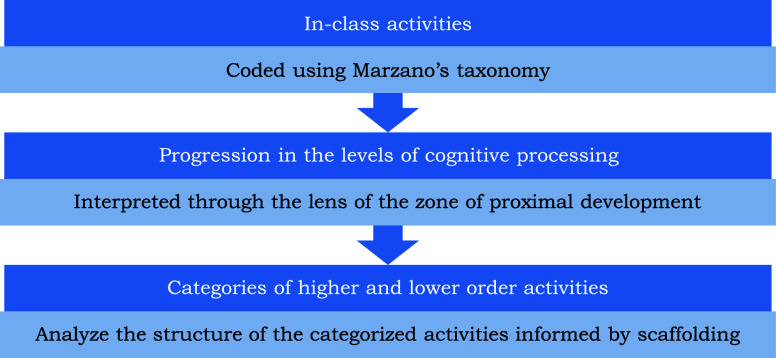
Visual diagram representing the processes for coding activities, categorizing each activity using the theoretical framework of social constructivism (ZPD) and then expanding upon the role each category of activities may play during the learning process.

Vygotsky’s theory of social constructivism and, more specifically, the Zone of Proximal Development (ZPD) guided this study. The constructivist and social constructivist views of learning are that students build their understanding through experiences, ideas, and social interactions.^36,37^ ZPD emphasizes the presence of a knowledgeable other in these interactions and describes a zone where the learner’s current level of knowledge cannot extend further without support from that more knowledgeable other.^36^ The knowledgeable other was originally defined to be anyone with more expertise regarding the subject, but building on the work of Wood and Ross,^8^ the knowledgeable other could be described by the six functions or support they provide to the learner. This expansion of the knowledgeable other allowed for researchers and faculty to implement and describe supports for students that are not dependent solely upon a single person. When related to Marzano’s taxonomy, the role of the knowledgeable other can now be supplemented by ensuring students are incrementally engaging in the cognitive levels of processing leading to higher-order questions. The functions of reducing the degrees of freedom and maintaining direction can be accomplished by asking questions that require students to retrieve relevant knowledge and identify critical features of that knowledge that are needed to answer later questions. In Figure 1, a diagram visualizing the method for coding and analyzing the activities can be seen. The extension of ZPD informs the work by providing a theoretical lens to categorize each activity based on the progression in the cognitive levels of processing and how that structure can support the students in expanding their ZPD.^38,39^ The categorized activities are then expanded upon by the possible role they may play during facilitation or the additional support a student may need while working and are informed by the theory of scaffolding.

### Workshops

The in-class activities assessed were developed by faculty who attended a multiday PALAC workshop in 2017 or 2018. The four workshops, which lasted three to 4 days, were held in person and facilitated by experienced analytical chemistry faculty. The workshops were designed to provide instructors with the tools needed to use active learning strategies within their courses and support them in designing active learning materials. Participants engaged in a series of modules, such as introducing active learning, developing learning objectives for their courses, and grant writing. These modules allowed participants to practice implementing active learning strategies through tasks such as defining and writing effective learning objectives, assigning Marzano’s levels of processing to example questions, and writing their own questions that corresponded to the levels of processing, culminating in the participants developing and modeling their own course activities. For a more detailed description of the workshops, see the work of Brown et al.^34^

### Participants

The 2017 and 2018 PALAC workshops had 40 and 42 participants, respectively, with no overlap in the workshops attended by participants. Analytical chemists from institutions across the United States and Puerto Rico were invited to apply to attend the workshops.^34^ Participants were selected based on factors intended to maximize the impact of the workshop, such as permanence of position, size of classes, prior experience with active learning, and previous experience teaching. Although the PALAC project was developed for analytical chemists, this was primarily focused on the faculty’s identity as an analytical chemist and not limited to instruction in analytical chemistry courses. Consequently, the course materials collected for analysis were from a broad range of courses and not just analytical chemistry. Additionally, these materials consisted of both prewritten materials that were then modified by the participant during the workshop and materials written during the workshop and during the subsequent semester. Of the 82 participants, 75 consented to have their data used in the research project, with 56 (74.7%) submitting materials suitable for this study. The study was approved by an Institutional Review Board (IRB), and all participants who submitted materials have been assigned pseudonyms to maintain confidentiality.

### Analysis

The collection of course materials spanned two years after the conclusion of the workshops and included a range of materials, including laboratory experiments (*n* = 46), quizzes/exams (*n* = 25), and in-class activities (*n* = 458), totaling 529 course materials. Given the goal of assessing the structure of in-class activities designed to support student learning in the classroom, only the in-class activities were selected for this analysis. Some of the course activities submitted included workbooks with multiple units and were separated into distinct activities for sampling and coding. Some of the course materials submitted were presentations that would be given during a lecture period. If the presentations involved questions that students were given time to answer in class, they were included in this study; those that did not ask questions of the students nor indicate specifically that students were to work on the problem (via a timer present or written instruction) were not included in the analysis.

The in-class activities analyzed were sampled from the 458 classroom activities using a stratified random sampling method.^40^ The stratified random sampling was used to ensure that every participant who submitted in-class activities was represented in the analysis by including activities from each of them. For any participant that submitted ten or fewer in-class activities, two activities were randomly sampled, and for participants that submitted 11 or more in-class activities, a 20 percent sampling was used, resulting in 134 in-class activities sampled across the 56 participants.

MAXQDA 2020 (VERBI Software, 2021) was used to assign a cognitive level to each question. Before coding the sampled activities, researchers coded activities not included in the sampling to familiarize themselves with the taxonomy. No alterations were made to the taxonomy, but we discovered that it was important to note the course each activity would be presented in. The prerequisite knowledge a student may have in general chemistry differs from that of a student in analytical chemistry, so the researchers accounted for this while coding the sampled materials. A system of assigning question numbers was implemented to ensure the activities were analyzed consistently. Questions that contained multiple parts were coded with a single level of the taxonomy if they were written within a singular block of text. If two related questions were asked but given clear separation through a vertical space between the questions, then these questions were coded separately. Marzano’s taxonomy describes the levels of processing as a hierarchical structure, so a question with multiple parts in a singular block of text is coded according to the highest level of processing because if a student is engaging in analysis (level 3), they are also engaging in the retrieval and comprehension levels of processing (levels 1 and 2).^16^ Whereas, for questions that had a clear separation between the previous question and the current one, it was important to code both questions to track how the level of processing changed. Going forward, questions that are denoted with 1a, 1b, 1c, etc., are questions that were related but clearly separated.

**Figure 2:**
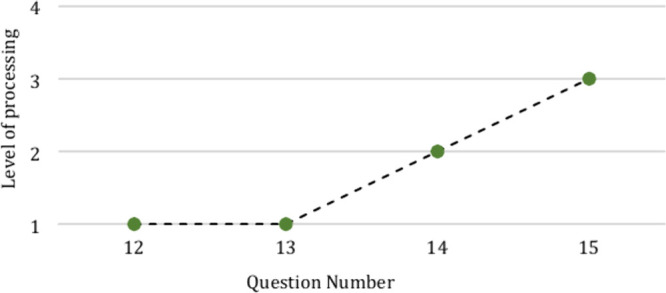
Marzano’s level of processing for questions 12–15 asked in a sample classroom activity.

Box 1 provides an example of questions coded for a portion of one of the activities, and Figure 2 provides the analyzed activity by plotting the progression in the levels of processing for each question.

Box 1 **Example questions from a worksheet submitted by RachaelD**

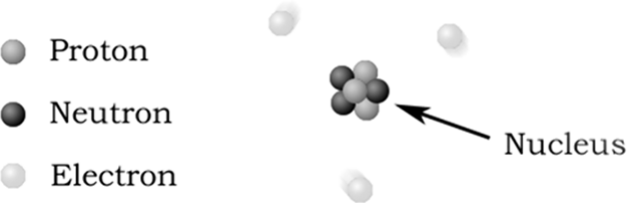

12. How many neutrons are in the model above? ___ electrons? ___ protons? ___

13. Which particles make up the nucleus (the “center” of the atom)?

14. The nucleus is referred to as a dense part of the atom that contains most of the mass of an atom. What does this tell you about the masses of protons and neutrons as compared to the mass of electrons?

15. After examining the model, which particle do you expect to be easiest to remove and why?

In question 12 of the worksheet, the students are asked how many neutrons, electrons, and protons are in the provided model. The question requires the students to identify the information from the image but provides a key for which circles are neutrons, protons, and electrons, meaning that the students just need to count the appropriate circles to arrive at the correct answer. The retrieval level of processing best describes this question since it simply requires students to recognize the number of each subatomic particle. Providing the students with a key that identifies the parts of the atom also aligns with the support of *marking critical features* and *reduction in degrees of freedom*. The same logic is applied to question 13, where the students need to identify the particles that make up the nucleus. Given the added help provided in the question “(the “center” of the atom)” and the large arrow labeled nucleus, the question requires that the students recognize information already provided to them in the model and was coded as a retrieval level of processing. In question 14, the students are asked to find an answer that cannot be derived from the model provided. The question requires that the student begin to consider not only which particles make up the nucleus but why it is regarded “as a dense part of the atom that contains most of the mass of an atom,” requiring the students to identify critical features of the atom. The emphasis on critical features is what leads to the question being coded as a comprehension level of processing. Question 15 then asks the students about which particle they expect to be easiest to remove. The question requires that the students retrieve information about what particles make up the atom and then think critically about the location and mass of each type of particle. Where questions 12 and 13 provided the support of *marking critical features* and *reduction in degrees of freedom*, question 15 now removes these supports to promote the students generating new insights. In relating the concepts of what subatomic particles make up the atom and density in the previous questions, the students are generating the new insight of ionization, and the question is coded as an analysis level of processing.

During the activity coding process, inter-rater reliability (IRR) was determined using the Kappa statistical analysis to ensure the researchers consistently interpreted the cognitive level of the questions. Twenty percent of the sampled activities were selected for IRR (*n* = 27), and a final value of 0.80 was calculated, indicating strong agreement.^41^ After the coding process was completed, plots were made for each of the activities to better visualize the change in the cognitive levels across the questions. The visual representations for the levels of processing provide insight into the general structure of the individual activities but given that the in-class activities covered a broad range of topics, assessing the structure needed to be independent of specific content areas. The analysis was based on the structure of the cognitive levels of processing leading to higher-order questions as cognitive structuring can reduce the possibility for cognitive overload.^20−23^ Just as ZPD describes the gap between a student’s current abilities and their possible abilities, effective support requires that the size of the gap be considered to adequately support learning. Too large of a gap between what the student knows and what they are meant to achieve can impede their learning.^8,36,42^ When applied to Marzano’s taxonomy, this idea is presented in how the cognitive levels change through in-class activities. Therefore, the activities that included higher-order questions were separated from those that only had lower-order questions (retrieval and comprehension levels of processing) to further analyze the cognitive structuring.

## Results and Discussion

Of the 134 activities sampled, 38.8% (*n* = 52) contained only lower-order questions, and 61.2% (*n* = 82) contained higher-order questions. This aligns with one intended goal of the PALAC workshop: to engage students in learning that extends beyond the memorization approach. Questions that engage students in higher-order thinking are indeed beneficial, but analysis of question level alone does not account for the cognitive structure of the activities.

**Figure 3:**
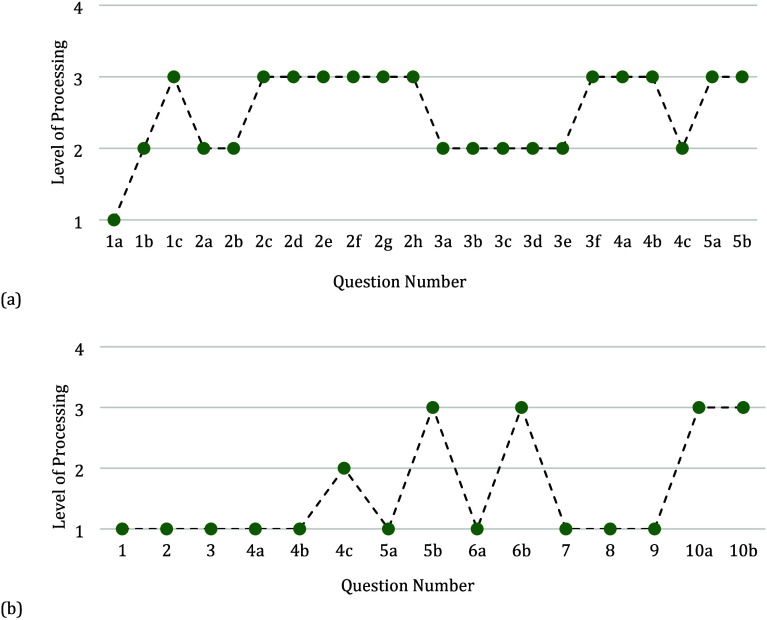
Marzano’s levels of processing for questions asked in the classroom activities. (a) Representative example of a classroom activity with continual progression from low-order questions to higher-order questions. (b) Representative example of a classroom activity that demonstrates a different type of progression from lower-order questions to higher-order questions.

With the lens of ZPD, in-class activities should ensure that the prerequisite knowledge is established before engaging the students in higher-order questions that expand upon that knowledge, thereby supporting the expansion of the student’s ZPD. When learning a new topic and working on an in-class activity, asking only analysis level questions provides no support in establishing the prerequisite knowledge needed to engage with the higher-order thinking described by the analysis level of processing and would require careful facilitation by the instructor. Consequently, effective support embedded in the materials would contain low-order questions to establish the necessary knowledge to then be applied to higher-order questions, thereby ensuring any task given is within the student’s ZPD.^20^ Examples of the different progressions in the levels of processing that lead to higher-order questions can be seen in Figure 3.

The worksheet illustrated in Figure 3a starts with a clear progression from low-order to higher-order in questions 1a, 1b, and 1c. Questions 1a and 1b serve to prime the prerequisite knowledge of the relationship between energy, frequency, and wavelength and then consider the critical features of these relationships by drawing energy diagrams for absorption and fluorescence. This knowledge is then applied to question 1c where students are given gas and liquid absorption data and asked to discuss and explain the differing features, representing an expansion of their understanding through a novel example that was supported by questions 1a and 1b. Figure 3b demonstrates jumps in the cognitive level of processing, specifically in questions 5a–6b, where the students are asked to identify just the number of peaks in a set of mass spectrometry data (questions 5a and 6a) and then asked to interpret and explain why they see these peaks in the data (questions 5b and 6b). Although both examples in Figure 3 ask higher-order questions, Figure 3b represents a different type of cognitive structuring given the level of processing preceding some of the higher-order questions do not engage the students in the comprehension level of processing, where the students are given the opportunity to identify critical features and practice moving their knowledge from permanent to working memory and vice versa. Therefore, in the following analysis, the sampled activities were categorized by the presence of cognitive support leading to higher-order questions to draw further conclusions.

### Activities with Higher-Order Questions

**Figure 4:**
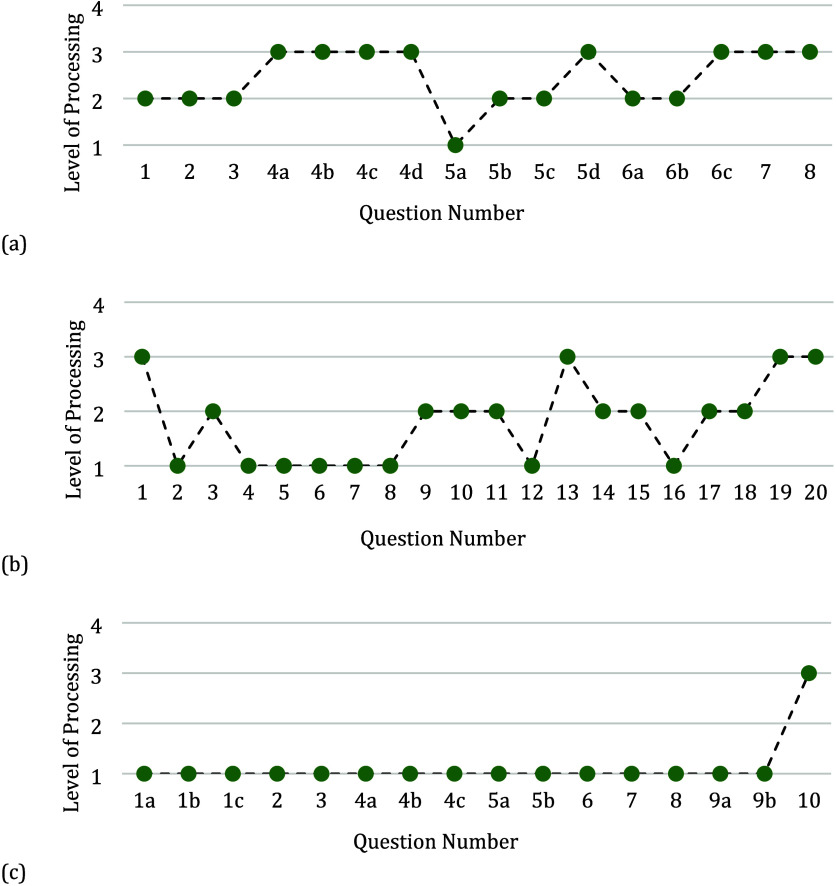
Marzano’s level of processing for questions asked in the classroom activity. (a) Representative example of a type 3 higher-order classroom activity where every higher-order question is preceded by a change in one level of processing or the same level of processing. (b) Representative example of a type 2 higher-order classroom activity where some of the higher-order questions are preceded by a change in one level of processing or the same level of processing. (c) Representative example of a type 1 higher-order classroom activity, where none of the higher-order questions are preceded by a change in one level of processing or the same level of processing.

For activities where all high-order questions were preceded by a change in one cognitive level (n-1) or by the same level, the activity was categorized as a type 3 higher-order activity (Figure 4a). If only some of the higher-order questions were preceded by a change in 1 level (n-1) or the same level, then they were given the category of a type 2 higher-order activity (Figure 4b). For activities that did not have any higher-order questions preceded by a change in one level or by the same level, they were placed in the type 1 higher-order category (Figure 4c). For each type of higher-order activity, this method of categorization was done based on the initial higher-order question. When multiple higher-order questions were asked in a sequence, the researchers defaulted to the structure leading to the initial higher-order question and categorized the activities accordingly. This method of characterizing is beneficial for researchers and faculty because the degree of cognitive scaffolding and structure is determined by the support provided to students through the level of processing and priming of prerequisite knowledge as they build to higher-order thinking.^43^ For example, if the students are not provided enough support, then higher-order thinking becomes difficult due to a high cognitive load that can be brought about by irrelevant information that affects working memory performance.^22,44^ By implementing cognitive structure we can provide students the opportunity to recall relevant information and identify critical features of that information to aid in transferring knowledge from working memory to permanent, thereby reducing the irrelevant information and cognitive load before engaging in higher levels of processing. Type 3 higher-order activities embed the support needed for students within the structure of the activity, whereas type 2 and 1 higher-order activities may require facilitators to provide additional support due to the reduction in cognitive structuring.

The worksheet in Figure 4a demonstrates that every higher-order question is supported by the lower levels or the same levels of processing where the prerequisite knowledge for the topic is addressed, thereby ensuring the students’ cognitive load is reduced.^23,45^ In questions 1–3, the students are asked to sketch a diagram of a flame-atomic absorption (AA) instrument and then asked what components of flame-AA are modified for graphite furnace-AA and inductively coupled plasma-atomic emission and how this changes the detection limits and allows for different atomic emission spectroscopy. Questions 1–3 have the students identify the critical features of the instruments, which is then used in questions 4a–4d where the students are provided with different sets of data (low concentration vs highly variable concentrations etc.) and asked which atomic spectroscopy technique would be best suited for each type of data. The first three questions prime the prerequisite knowledge the students need for question 4, consequently reducing the cognitive load of the higher-order questions (4a–4d).

The worksheet shown in Figure 4b represents a type 2 higher-order activity and differs in that question 1 is a higher-order question (analysis level of processing) asking the students to describe a procedure that accounts for the matrix effect on measurements. In doing so, the activity does not provide a priming of the prerequisite knowledge for students and may require additional support from the facilitator. Asking a higher-order question with no support aligns with the idea of fading scaffolding to assess the student’s understanding of a previously taught topic, but facilitators must be aware of students’ likely prerequisite knowledge and the possible difficulties students may have. This worksheet does provide support to higher-level questions, specifically in questions 16–20 where students are asked questions about the use of an internal standard, the drawbacks of one point calibration, and to describe a method for using an internal standard before the final questions give two case studies to analyze for errors. The attention to the students’ cognitive processing in the later questions implies the activity was developed with the progression of learning in mind and therefore serves to reduce the amount of support required from the facilitator.

When these examples are juxtaposed with the type 1 higher-order activity shown in Figure 4c, the difference in the structure of activities and the level of processing becomes apparent. Questions 1a–9b are all retrieval and only ask calculation questions about determining concentrations of diluted solutions until question 10, which asks the students to write a short paragraph that describes how to prepare 100.0 g of a 5.0 wt %. aqueous solution of sodium chloride. Although questions 1a–9b can represent a degree of priming the prerequisite knowledge, such as how to perform a calculation, the worksheet lacks cognitive structuring to link the calculations to the more cognitively demanding task of planning out a dilution and, therefore may require the most amount of additional support from a facilitator when compared to the type 2 and 3 higher-order activities. A possible remedy for the lack of cognitive structuring might be to add a comprehension question before the analysis question where the students are asked to identify the critical features needed to prepare a dilute solution such as the type of dilution and the physical states of the solvents/solutes being used.

The distinction between how the knowledge is being used and processed at each level demonstrates the benefit of students engaging with comprehension before moving on to the analysis level of processing while learning. In doing so, the students are afforded the opportunity to practice moving knowledge between permanent and working memory, identify critical features, and practice incorporating the concepts into their current knowledge structure. Thus, the students are supported during the learning process with a *reduction in degrees of freedom*, *direction maintenance*, and *marking critical features* as described by Wood and Ross^8^ When the students engage with a retrieval-level question and then an analysis-level question, they are not given as much support in moving the knowledge between permanent and working memory as they engage with the analysis-level question. Consequently, when viewing the example in Figure 4c through the lens of ZPD, there is less support for the students as they expand and incorporate their new understanding of the concept, and may require additional support, such as a *demonstration*, from an outside source. From these examples, the importance of considering the students’ cognitive abilities becomes apparent and the types of higher-order activities serve to demonstrate the level of support provided and the possible need for external support from the facilitator. The breakdown of the different types of higher-order activities along with their percentages of the total activities sampled can be seen in Table 2.

**Table 2:** Number of Activities in the Type 1, 2, and 3 Higher-Order Categories and the Percentage of the Total Number of In-Class Activities Sampled

	Type 1 higher-order	Type 2 higher-order	Type 3 higher-order
No. of activities in each higher-order category (*n* = 82)	15	25	42
Percent (%) of total (*n* = 134) activities	11	19	31

Of the 134 activities, 61.2% (*n* = 82) contained higher-order questions, while only 31.3% (*n* = 42) demonstrated continual support leading to higher-order questions. The increase in the percentage of total activities across the type 1, 2, and 3 higher-order categories in Table 2 implies that the workshop was successful at getting faculty to value the benefit of higher-order thinking. This is further supported when considering that these participants likely had little experience with active learning methods and that 31 of the 56 (55%) participants submitted activities that all contained higher-order questions. However, the percentage of type 1 and 2 higher-order activities demonstrates that more attention may need to be given to how professional development provides support that aligns with the theories of scaffolding and ZPD outside of just cognitive structuring.

### Activities with Only Lower-Order Questions

**Figure 5:**
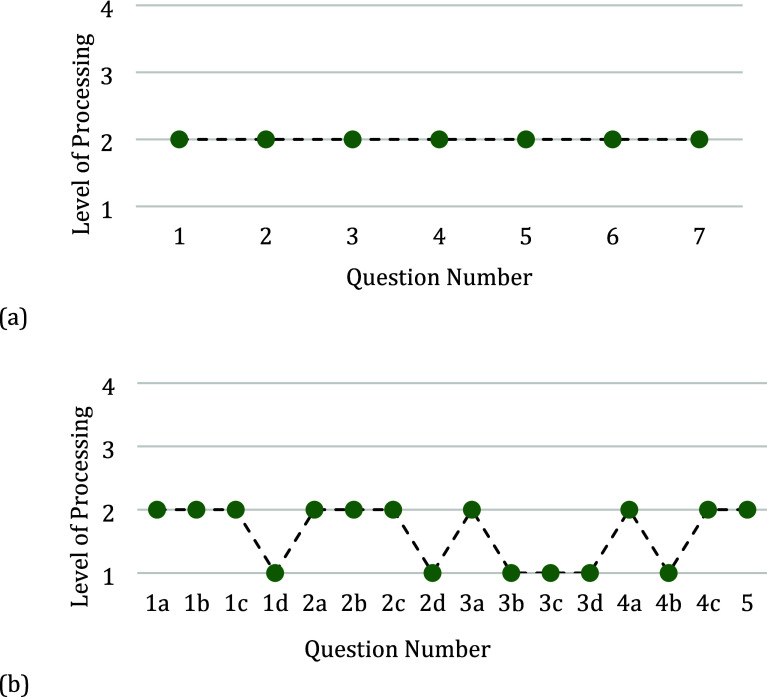
Marzano’s level of processing for questions asked in the classroom activity. (a) Representative example of the lower-order 1 category of classroom activities where the questions only contained a single lower-order level of processing. (b) Representative example of the lower-order 2 category of the classroom activities where the questions contained both retrieval and comprehension levels of processing.

Activities that did not contain any higher-order questions were categorized based on the presence of containing only a single level of processing for all questions (lower-order 1) (Figure 5a) or a mix of both retrieval and comprehension (lower-order 2) (Figure 5b). For the lower-order 1 activities, in all but one case these activities contained just comprehension questions. Table 3 provides a breakdown of the number of activities in each category along with their percentages of the total activities collected.

**Table 3:** Number of Activities in the Lower-Order 1 and 2 Categories and the Percentage of the Total Number of In-Class Activities Sampled

	Lower-order 1	Lower-order 2
No. of activities in each lower-order category (*n* = 52)	25	27
Percent (%) of total (*n* = 134) activities	19	20

Of the 134 in-class activities sampled, 38.8% (*n* = 52) did not ask any higher-order questions, indicating the workshop was not entirely successful at getting faculty to implement higher-order thinking let alone cognitive structuring. The absence of higher-order questions represents more of a rote learning approach, often seen in homework questions, where students are asked to retrieve facts and perform calculations. The lower-order activities may represent a more traditional approach to questions and a lack of adopting the active learning strategies demonstrated in the workshop. When viewed through the lens of ZPD and scaffolding, there is the opportunity for these activities to be used as a support for priming prerequisite knowledge that can be expanded during another activity or in a lecture, but dedicating significant in-class time to these types of activities distracts from the goal of engaging students in higher-order thinking. Consequently, it is important to consider that this study analyzes the worksheets, not the course itself. It is not the position of this paper to comment on the scaffolding of a course, just the structure of the activities themselves. Regardless, the number of lower-order activities does indicate that there needs to be more support for faculty in how to design and achieve the goal of getting students to engage in higher-order thinking while providing cognitive support.

## Conclusions

During the PALAC workshop, participants completed modules on using Marzano’s taxonomy, with the learning objective of these modules being to develop activities that used scaffolding to support student’s engagement with higher-order thinking. In-class activities collected from faculty who attended the PALAC workshop were analyzed for their cognitive structuring using Marzano’s taxonomy to classify the cognitive level of each question. The analysis found that 61.2% (*n* = 82) of the activities engaged students in higher-order thinking, which implies the successful adoption of the approach presented in the workshop. Upon further analysis and categorization of the lower and higher-order activities, the findings appear to be more nuanced, and a summary table of the number of activities and their percentage of the total in-class activities sampled can be seen in Table 4.

**Table 4:** Number of Activities in the Lower-Order (1 and 2) and Higher-Order (Type 1, 2, and 3) Categories and Their Percentage of the Total Number of In-Class Activities Sampled

	Lower-order 1	Lower-order 2	Type 1 higher-order	Type 2 higher-order	Type 3 higher-order
No. of activities in each lower-/higher-order category (*n* = 134)	25	27	15	25	42
Percent (%) of total (*n* = 134) activities	19	20	11	19%	31

Previously, we discussed the criteria for selecting participants to attend the workshop, which was based on the time spent teaching and experience with active learning. The reason for this was to have participants who had likely not been exposed to active learning methods and provide them with an opportunity to learn and practice these methods. As we can see in Table 4, the majority of sampled activities fall into the type 2 and 3 higher-order categories (50%), implying that the workshop was successful at getting faculty to adopt the desired teaching practices. However, when considering the lower-order 1 and 2 categories represent 39% of the sampled activities, where no higher-order question is asked, there remain opportunities for additional faculty development to demonstrate the importance of higher-order thinking. Given these findings, faculty development projects would benefit by ensuring participants understand the benefits of higher-order thinking and providing sustained support to faculty through check-ins and the development of a community of practice.

It is important to note once again that this analysis considers just the cognitive structure and not the scaffolding of a course. We use scaffolding to describe the types of support the workshop aimed to instill in the participants but to effectively scaffold a course; one must consider how to support student learning throughout a course and not just while working on an activity. Consequently, activities in the type 1 and 2 higher-order categories, which are those that either provided no cognitive structuring to higher-order questions or provided it to only some of the higher-order questions, may serve a purpose in evaluating a student’s ability to engage with higher-order thinking when cognitive support is not present. Similarly, the lower-order activities, which did not ask a single higher-order question, could have a place in courses as a priming of prerequisite knowledge before engaging in higher-order thinking. Future studies would benefit from considering this possibility to better understand the role of cognitive structuring and facilitation in developing a scaffolded learning environment.

These findings demonstrate how Marzano’s taxonomy can be used to aid faculty and researchers in developing and understanding scaffolding by considering the levels of cognitive processing being engaged with by students during the learning process. The findings from this study also provide insight into possible improvements that can be made in faculty development workshops, such as providing more time to practice implementation of active learning strategies across entire in-class activities during the workshops or providing check-ins with participants after the conclusion of the workshop to ensure consistent implementation.

## Limitations

This work was designed to specifically assess the structure of classroom activities and the cognitive support they provide to students. While scaffolding can be used as a tool to ensure students are provided this support, it is important to note that effectively scaffolding a course requires considerations to be made for all aspects of learning and not just the activities.^46^ Therefore, when considering the insights provided in this work, researchers and faculty must be aware of the purpose of classroom materials and how they can be leveraged to aid in the learning process. For this study, the researchers performed their analysis based on the activities collected and did not have data regarding how or when these in-class materials were used. When students are being introduced to a new topic or concept, a higher degree of cognitive structuring can serve to support learning, as seen in type 3 higher-order activities, but as students gain competency in said topic the cognitive structure should begin to fade, transferring the responsibility to the students.^47^ This project was able to demonstrate how Marzano’s taxonomy can be used in conjunction with scaffolding but future work in the field would benefit from consideration of when these activities are presented to students during the learning process.

In tandem with the consideration given to the role of course activities in scaffolding learning, it is important to note that the levels of processing defined by Marzano are described from the perspective of the learner. Meaning that a question written by faculty to engage students in the comprehension level of processing may not elicit the desired level of processing in the student. Such a limitation corresponds to the first point made in that researchers and faculty must consider not only the role of course activities but also how the students engage with them. Determining the level of scaffolding can be impacted by the environment and facilitation of such content. The assigned level of processing acts as a proxy and the actual level of processing for each question may differ depending on the guidance provided by the facilitator or other outside resources. Similarly, the analysis of the materials for this research included the breaking up of questions depending on the space between each and may have led to a mismatch in what the faculty who developed the in-class activities intended and how they were analyzed. Such a limitation is mitigated due to the hierarchical nature of Marzano’s taxonomy but is still important to consider for implementation and future research.

## Implications

### Instruction

The results from this project indicate that Marzano’s taxonomy can be used to assess the structure of activities and the support they provide for student learning. For example, if a lecture period is meant to expand on a topic previously covered, then a quick worksheet at the start of the lecture that contains retrieval and comprehension level questions can help prime the prerequisite knowledge needed to further develop understanding, while a worksheet containing both low-order and higher-order questions structured properly can aid in expanding the student’s understanding. While not limited to worksheets, this method of developing scaffolds with Marzano’s taxonomy can be applied to lectures, homework, projects, exams, and the structure of a course overall.

### Faculty Development Projects

Faculty development projects are no easy undertaking, but improvement requires identifying strengths and weaknesses to serve as indicators for how future projects can be better developed to help faculty implement active learning strategies. In the case of the PALAC project, time was given for the participants to practice writing learning objectives and questions that aligned with the levels of processing described by Marzano, but not as much time was spent developing an understanding of the complex process of structuring the levels of processing across entire worksheets or how the different types of higher-order activities can effectively be used. Faculty development projects would benefit from devising means for continuing to support participants after the conclusion of the workshop. Therefore, the results of this study suggest that faculty development projects should focus more on how active learning strategies are implemented in courses, how course materials can be structured to support learning, and provide a means for feedback on activities after the conclusion of the workshop.

### Research

Research regarding scaffolding is difficult due to the dynamic nature and complexity of the learning environment with the primary challenge being how to measure scaffolds.^48^ Effective scaffolds require an understanding of where and when the students have difficulty processing information, and the application of Marzano’s taxonomy can provide insight into those difficulties. Furthermore, the application of Marzano’s taxonomy affords another avenue for assessing the impact of facilitation on student learning. By developing activities structured around the cognitive levels of processing, researchers can analyze student data and determine when the intended level of processing differs from that enacted by the students and why. The field of chemistry requires students to consistently change how they view matter and processes, from macroscopic to submicroscopic and symbolic, and understanding how students process these transitions cognitively can aid in further developing researchers’ understanding of student learning in chemistry. This article serves to establish how Marzano’s taxonomy can be used to evaluate and construct scaffolding, but future studies can expand upon it and would benefit from analyzing the role of external factors in student learning in conjunction with comparing cognitive structures impact on student discourse in group learning.

## Supporting Information Available

Expanded codebook for Marzano’s levels of processing and an activity with applied codes/reasoning for the code (PDF, DOCX)
()
