# Supplementary material for: Structuring Materials to Support Student Learning: Analysis of Instructional Materials from a Professional Development Workshop
Source: J Chem Educ. 2024 Sep 30;101(11):4603–13. doi: 10.1021/acs.jchemed.4c00783 (PMC11562375; doi:10.1021/acs.jchemed.4c00783)
Supplement: Supplementary file 2 — ed4c00783_si_002.docx [file ed4c00783_si_002.docx]

Structuring materials to support student learning: Analysis of instructional materials from a professional development workshop

Authors: Andrew Kreps^1^_,_ Ian Brown^1^, Thomas J. Wenzel^2^, and Renée Cole^1^

^1^Department of Chemistry, University of Iowa, Iowa City, IA 52242, USA
^2^Department of Chemistry, Bates College, Lewiston, ME 04240, USA

**Supporting Information**

**Table of Contents**

Full codebook for Marzano’s level of processing ………………………………Pages 2 – 7

Example activity with reasoning for the coded level of processing……….…Pages 8 – 11

**Full codebook for Marzano’s level of processing**

| **Marzano Cognitive Level of Processing** | **Subcategory** | **Definition** | **Key Features** | **Example** |
| --- | --- | --- | --- | --- |
| Retrieval - Recalling information from permanent memory | Recognize | Finding indicated information in provided materials | Prompt specifies what information needs to be found Source material is provided | What scan rate did the authors use for the analysis? |
|  | Recall | Reiterating explicitly covered material in a closed question type | Prompt addresses material previously covered in the course or expected to be addressed in prior courses Answer choices are provided | Which of the following represents a cation? A. Ti B. 14C C. Na+ D. S2- |
|  | Recall | Reiterating explicitly covered material in a open question type | Prompt addresses material previously covered in the course or expected to be addressed in prior courses Free response answer | What is meant by an “internal standard”? |
|  | Executing | Completing an explicit, often algorithmic task. | Plug and chug exercises where students are doing rote calculations Task involves following a set of rules or steps/procedures | a) The density of titanium metal is 4.51g/cm3 at 25 °C. What mass of titanium displaces 125.0 mL of water at 25 °C b) Weight out and add 1 gram of sodium hydroxide pellets to the solution. Record the exact mass. |
| Comprehension - Identification of critical features needed to transfer knowledge from working memory to permanent. | Integrating | Identifying the important or relevant information from a larger pool | Task requires the main idea(s) to be extracted from a larger body of information The prompt either provides a body of material that contains more information than is needed to address the question or each response must be evaluated to determine which is relevant | a) Summarize the article in one sentence. b) Which of the following correctly describes the process of expiration (air leaving the lungs)? 1) There is no change in the internal pressure in the lungs. 2) The lungs expand, causing their internal pressure to increase 3) The lungs contract, causing their internal pressure to decrease, and attract air to the lung. 4) The lungs contract, causing their internal pressure to increase, and expel the air out of the lung. 5) The lungs expand, causing their internal pressure to decrease. |
|  | Symbolizing | Transforming information to or from graphical/visual representations. | Generate a graphical representation from data or generate a table/equation from a graph Transform a representation from one form to another (text to image, one schematic to another, etc) Convert between symbolic, microscopic, and macroscopic representations | a) Draw the energy diagram showing all the energy levels associated with E0 and E1 b) Draw a schematic that illustrates the steps of the sample preparation and analysis. |
| Analysis - Creating new insights from previous knowledge or using previously obtained knowledge in novel situations (focus is on the concept/skill). | Matching | Comparing and contrasting two or more things | Prompt requires students to evaluate similarities and differences of key features | Suggest some advantages of electrochemical detection of dopamine for these applications, compared to spectroscopic or mass spectrometric detection. |
|  | Analyzing Errors | Analyzing a question for errors, and if they exist, correcting them | Prompt explicitly tells students to look for and correct any errors | Individuals recovering from drug addiction are often counseled to avoid “triggers”, including locations and situations in which they previously used drugs. Do the findings in this study support this advice? Why or why not? Cite a specific figure or figures in your answer. |
|  | Generalizing | Deriving a broad pattern for specific examples | Extract a pattern from examples | Why does it matter where/when we collect our samples? (Random vs. Judgmental vs. Systematic, Convenience, or combinations of these) |
|  | Specifying | Applying general patterns to novel situations or questions | Task situated in a context not covered in course materials Apply explicitly covered patterns to make predictions | Given the following four compounds to be separated on a C18 column with an isocratic elution, predict the order of elution and justify your answer. |
| Knowledge Utilization - Using knowledge for novel and specific situations (focus is on the situation) | Problem-Solving | Identifying issues, and planning and executing a solution to **unexpected issues.** | Task has multiple valid solutions Involve more authentic contexts Problem context requires resolving obstacles to the goal Justification of choices is often required |  |
|  | Decision-Making | Making a choice about how to proceed on a subject and justifying why that choice was made. | Task usually has multiple valid solutions Involve more authentic contexts Requires explanation for the choices made *Note: Often paired with Analysis tasks - the addition of justification places the task in Knowledge Utilization | Explain, with numerical values, glassware, and instruments how you would prepare 50 mL of a 1.40 M stock solution of sodium thiosulfate, Na2S2O3 (MW= 158.11 g/mol) Na2S2O3 (MW= 158.11 g/mol) while introducing the least amount of error. |
|  | Experimenting | Plan and execute an experiment to answer a question. | Typically involves hypothesis testing Expectation is that this is physically done not just hypothetical | Review the chemical literature and select an experiment that is of interest to you and that contains significant physical chemistry content. The Journal of Chemical Education is a good source of experiments you may want to pursue. You may also propose an extension of one of the experiments you have completed during the semester. The experiment you choose will serve as the subject for a four part independent project, although you may work with your lab partner(s), that includes a project proposal, experimental protocol, data report, and presentation. |
|  | Investigating | Synthesize the literature to determine the answer to a question. | Sources and relevant information are not identified  Information from multiple external sources is required Requires different types of information to be combined |  |

**Example activity with reasoning for the coded level of processing**

Discussion Activity 3

**THE PLAN:** Our big overarching goal is to figure out how enzymes work in extreme environments. Last week we talked about how enzymes can impact reactions in the body by acting as a catalyst. Today we are going to look further at how enzymes work. In this case we are not going to look at just the active site but expand this to look at protein folding and how that relates to entropy.

The learning objectives and skills for today’s session are for you to:

| **Learning Objectives** | **Skills** |
| --- | --- |
| Predict whether a reaction is energetically favored based on the relative strength of chemical bonds and intermolecular forces in reactants and products | List factors that determine energetic stability |
| Predict whether a reaction is entropically favored based on the state of matter, molar mass, and complexity of reactants and products | List factors that determine the number of configurations for the system (matter and energy) |
|  | Explain how/why factors affect entropy values |
|  | Compare systems in terms of the relative number of distinguishable configurations |
| Qualitatively predict the effect of changing temperature on reaction directionality based on the relative energetic stability of reactants and products | List factors that determine overall thermodynamic stability |

# PRE-CLASS QUESTIONS

Take the first few minutes of discussion to ensure that everyone agrees on the answers before moving on to the worksheet.

1. Define the following:
   1. Entropy

Level of Processing: Retrieval

Reasoning: Students only need to recall information or are given with the video.

- 1. Intramolecular forces
  2. Intermolecular forces

1. List the intermolecular forces discussed in lecture.
2. Watch the following video that discusses protein folding and list the four levels of protein folding. Understanding the levels of protein folding will be helpful for the rest of the worksheets, where we will unpack the forces involved in protein folding. <https://www.youtube.com/watch?v=hok2hyED9go>

# QUESTIONS

In the last discussion we explored how the strength of a bond between atoms in a molecule (intramolecular forces) can affect the enthalpy change of a reaction and this week we will build from this idea but focus on the role of the forces between different molecules or parts of the same molecule (intermolecular forces).

1. Sketch a simple picture of both a folded and an unfolded protein. This can be represented as just a line.

Level of Processing: Comprehension

Reasoning: In this question, the students are transforming information into a visual representation.

1. Energetic factors focus on the potential energy of the substance due to interactions between submicroscopic components (i.e. electrons, atoms, ions, molecules). For protein folding, energetic factors are related to intermolecular forces. What type or types of intermolecular forces are involved in protein folding for the protein chain shown below? Explain the reasons for your choice(s).

Level of Processing: Comprehension

Reasoning: In this question, the students identify important information from a larger pool and transform the information from the figure.

1. As the protein folds, interactions occur between the different parts of the protein chain to optimize intermolecular forces within the protein itself and with the solvent.
   1. How would this folding affect the enthalpy of the protein? Justify your answer.
   2. How would the folding affect the entropy of the protein? Justify your answer.

Level of Processing: Analysis

Reasoning: Here the students are linking the concepts of enthalpy and entropy to the novel situation of protein folding.

1. Thinking about your team’s answers to question 3, discuss with your group the folding of the protein and decide together if folding makes it more or less stable. Be sure to explain your team’s reasoning in terms of the energetic and entropic factors.

Level of Processing: Analysis

Reasoning: Similar to the previous question, this question now has the students develop new insights by connecting the concept of stability to the novel situation of protein folding.

1. Thinking about your team’s answers to question 3 …
   1. Draw a PEC diagram that represents the relative potential energy and number of configurations for the unfolded and folded protein.
   2. Explain which factor(s) provides the driving force(s) for protein folding.

Level of Processing: Comprehension

Reasoning: In this question, the students transform information into a visual representation and require that they identify the critical features of the PEC diagram.

1. Up until this point we have discussed the forces within the protein chain that drive it to fold, but one thing we haven’t considered is the environment surrounding the protein. Open the simulation on protein folding found on page 201 of your textbook or use the link below.

(<https://lab.concord.org/embeddable.html#interactives/samples/5-amino-acids.json>)

Notice that you can change both the solvent type and the hydrophobicity of the protein. Change the settings to “all hydrophobic.” (Reminder: Hydrophobic means that the molecule does not interact or “like” water and Hydrophilic means that it does)

- 1. Before running the simulation, talk with your group and come up with a prediction for what you think will happen to the protein if you set the solvent to water. If your team cannot come to a consensus, that is okay; just be sure to discuss all the predictions and write them down along with a justification for the prediction(s).
  2. Run the simulation and explain if your prediction was correct. If it wasn’t, discuss the results with your group and explain how it was incorrect.

Level of Processing: Analysis

Reasoning: For this question the students must expand their understanding of a topic by applying their understanding to a novel situation and derive a behavior from the example.

## **QUICK REFLECTION**

In question 6, your group needed to discuss and come up with a prediction for the simulation. During this, your team engaged in the skill of interpersonal communication, a skill that is incredibly important while working with others. Every one of us can probably think of a time when we worked with someone that either didn’t listen or didn’t acknowledge the contributions of others and that only makes working together more difficult, so let’s take a minute to reflect on how your team did to help us improve our communication skill. Read through the three characteristics on the left with your group and assign a number for each.

| **Interpersonal Communication** | | | Exchanging information and ideas through speaking, listening, responding, and non-verbal behaviors | | | |
| --- | --- | --- | --- | --- | --- | --- |
| No evidence | Rarely |  | Sometimes |  | Consistently | **Suggestions for Improvement:** |
| 0 | 1 | 2 | 3 | 4 | 5 | Restate or write down what was communicated. |
|  | Used a tone when speaking that invited other people to respond | | | | | Use a tone that is respectful and encouraging rather than confrontational or harsh. |
|  | Referenced others’ ideas to indicate listening and understanding | | | | | State what others have said in your own words and confirm your understanding. |
|  | Rephrased or referred to what other group members have said | | | | | Ask a follow-up question or ask for clarification. |

Now, discuss with your group and pick one specific characteristic your group can improve on and explain why you picked this characteristic. Lastly, circle the suggestions for improvement you think are applicable based on your group’s discussion.

# CONTINUING ON…

1. Now change the simulation settings to water and mostly hydrophilic amino acids.
   1. How does this change the protein folding process? What are the similarities and differences?
   2. Discuss with your group and provide a hypothesis to explain this observation.

Level of Processing: Analysis

Reasoning: For this question, the students must apply their understanding to a novel situation. Specifying the possible reason for the differences observed based on their understanding of entropy, enthalpy, and protein folding.

1. Looking at the simulation, how does your group think the hydrophobicity/hydrophilicity of the amino acids in an aqueous solution impacts the entropic factors?

Level of Processing: Analysis

Reasoning: Here the students are linking the concepts of entropy to the novel situation of hydrophobicity/hydrophilicity.
